# Comparative genomics of DNA-binding transcription factors in archaeal and bacterial organisms

**DOI:** 10.1371/journal.pone.0254025

**Published:** 2021-07-02

**Authors:** Luis Martinez-Liu, Rafael Hernandez-Guerrero, Nancy Rivera-Gomez, Mario Alberto Martinez-Nuñez, Pedro Escobar-Turriza, Eveline Peeters, Ernesto Perez-Rueda

**Affiliations:** 1 Instituto de Investigaciones en Matemáticas Aplicadas y en Sistemas, Universidad Nacional Autónoma de México, Unidad Académica Yucatán, Mérida, Yucatán, México; 2 Catedras-CONACyT, Instituto Nacional de Salud Pública, Cuernavaca, Morelos, México; 3 UMDI-Sisal, Facultad de Ciencias, Universidad Nacional Autónoma de México, Mérida, Yucatán, México; 4 Centro de Investigación Científica de Yucatán, Mérida, Yucatán, México; 5 Research Group of Microbiology, Vrije Universiteit Brussel, Ixelles, Belgium; University of Nebraska-Lincoln, UNITED STATES

## Abstract

Archaea represent a diverse phylogenetic group that includes free-living, extremophile, mesophile, symbiont, and opportunistic organisms. These prokaryotic organisms share a high significant similarity with the basal transcriptional machinery of Eukarya, and they share regulatory mechanisms with Bacteria, such as operonic organization and DNA-binding transcription factors (TFs). In this work, we identified the repertoire of TFs in 415 archaeal genomes and compared them with their counterparts in bacterial genomes. The comparisons of TFs, at a global level and per family, allowed us to identify similarities and differences between the repertoires of regulatory proteins of bacteria and archaea. For example, 11 of 62 families are more highly abundant in archaea than bacteria, and 13 families are abundant in bacteria but not in archaea and 38 families have similar abundances in the two groups. In addition, we found that archaeal TFs have a lower isoelectric point than bacterial proteins, i.e., they contain more acidic amino acids, and are smaller than bacterial TFs. Our findings suggest a divergence occurred for the regulatory proteins, even though they are common to archaea and bacteria. We consider that this analysis contributes to the comprehension of the structure and functionality of regulatory proteins of archaeal organisms.

## Introduction

Regulation of gene expression is a central process for all organisms in response to environmental changes [1]. Gene regulation includes diverse elements and mechanisms to allow or prevent the synthesis of specific gene products, such as transcription factors (TFs), small RNAs, or riboswitches, and structural elements associated with DNA and located in the intergenic regions, such as promoters and operators [1].

Since the description of Archaea as the third cellular domain [2], a large diversity of organisms, habitats, and life styles, such as mesophiles, extremophiles, opportunists, and symbionts, has been described [3]. In terms of regulatory mechanisms, archaeal organisms possess a basal transcription machinery resembling that of eukaryotes (consisting of up to 15 components) [4], such as a TATA box–binding protein (TBP), and diverse subunits of RNA polymerase, such as the F/E (RPB4/7) subunit, and a homologue of the transcription factor TFIIB [5]. In contrast, archaeal genomes encode bacterial-like DNA-binding TFs that function to repress or activate gene expression, by binding to DNA at specific sites around the promoter, allowing the regulatory machinery to express genes in response to different stimuli [6, 7]. These bacterial-like regulators usually contain a helix-turn-helix (HTH) DNA-binding domain and interact with a DNA motif of dyad symmetry, by using a dimeric structure. For instance, the ArcR protein regulates the genes devoted to arginine fermentation in the archaeon *Halobacterium salinarum*. This protein is homologous to negative regulators of transcription of genes of catabolic pathways, such as KdgR from *Erwinia chrysanthemi*, GylR from *Streptomyces griseus*, GylR from *Streptomyces coelicolor*, and IclR from *Salmonella typhimurium* and *Escherichia coli* [8]. Recently, the repertoire of TFs has been described for 52 archaeal genomes, suggesting they have different regulatory mechanisms than their bacterial counterparts and also exploring the hypothesis that archaeal TFs have multicomplex structures similar to as eukaryal ones [9].

In addition, the genetic organization of Archaea is structured into operons which are cotranscribed into common mRNAs in a similar way as bacterial mRNAs [9]. In this regard, diverse studies across almost all bacteria and even archaea have shown that operons are characterized by close spacing of genes within operons, modest conservation, and modest functional similarity [10]. However, in some archaea, the first genes of operons often have no 5′ untranslated region, so that the translation start is the transcription start [11, 12].

Comparative analysis of archaeal and bacterial genomes represents an opportunity to understand how similar archaea and bacteria are and to understand the evolution of gene regulation in prokaryotes. The present work aims to contrast the differences and similarities in the composition, structure, and function of TFs in these two cellular domains. To this end, we cover the challenge to identify TFs in archaeal sequence genomes, the identification of families common to archaea and bacteria, and a functional comparison between both datasets. In this work, TFs are defined as those proteins binding to DNA to activate or repress gene expression but that do not belong to the basal transcriptional machinery. We finish with some conjectures that attempt to provide a comprehensive picture of TFs in these organisms.

## Material and methods

### Archaeal genomes analyzed

A total of 415 genomes of Archaea and 12466 Bacterial genomes were downloaded from the NCBI Refseq genome database [13].

### Identification of DNA-binding TFs

The program pfam_scan.pl was used to scan the 415 archaeal genomes by using 16,712 hidden Markov models (HMMs) from the PFAM database [14]. An E-value of ≤10^−3^ was considered, with the option of clan_overlap activated (to show overlapping hits within clan member families). From these assignments, we extracted those proteins associated with 123 PFAMs described as DNA-binding TFs collected from regulatory proteins deposited in diverse databases, such as the DBD, RegulonDB, and DBTB databases, and those identified by manual curation (S1 File). In addition, the collection of 668 TFs with experimental evidence deposited in the Encyclopedia of TFs were compared against the complete set of 106 archaeal genomes, with an E-value of ≤10^−5^ and a coverage of ≥70% [15]. Finally, the set of TFs includes those proteins identified by PFAM and protein searches.

### Identification of catalytic activities

To determine whether TFs are associated with enzymatic activities, the Catalytic Families (CatFam) were used to scan the complete set of proteins associated with the 415 nonredundant archaeal genomes, using default conditions. CatFam generates sequence profiles to assign catalytic activities on protein sequences, minimizing the rate of false-positive predictions [16].

### Identification of TFs with virulence roles

To identify TFs with probable roles in virulence, a total of 3224 proteins retrieved from the Virulence Factor Database (VFDB) [17] were compared against the proteins of the 106 nonredundant archaeal genomes, with an E-value of ≤10^−5^ and a coverage of ≥70%, with the program Proteinortho. In a posterior step, we crossed the information of virulence homologous proteins identified in archaea with probable regulatory roles, as described in the previous section. VFDB considers proteins associated with experimentally verified virulence factors and includes proteins that may contribute to pathogenicity [17].

### Statistical analysis

Nonparametric Wilcoxon rank-sum statistical tests was used to evaluate proportion of TFs, enzymes and disorder regions in all the archaea versus bacterial genomes. Statistical significance was set at P ≤ 0.05. The PFAM domains identified were statistically analyzed to assess the existence of significant differences in their relative proportions. To carry out the statistical evaluation we used the Statistical Analysis of Metagenomic Profiles (STAMP) software 2.1.3 [18]. A White’s non-parametric t-test was implemented for hypothesis testing. A Benjamini-Hochberg False Discovery Rate (FDR) correction was applied on these data to identify statistically significant differential features among PFAM domains. Results with q-value < 0.05 (corrected p-value) were considered as significant and the biological relevance of the statistics was determined by applying a difference of at least 1% between the proportions.

## Results and discussion

### The repertoire of DNA-binding TFs exhibits a different distribution in Archaea than in Bacteria genomes

In order to identify those proteins devoted to regulation of gene expression in archaeal genomes, diverse bioinformatic tools were implemented. In brief, the prediction integrated Pfam searches and sequence comparisons considering the dataset of 668 well-known TFs as reference [7]. Based on this approach, we identified 40478 TFs in 415 archaeal genomes classified in two main phyla, Crenarchaeota and Euryarchaeota, and representing 14 orders. From the repertoire of TFs in those organisms, diverse observations emerged (S2 File).

**Fig 1:**
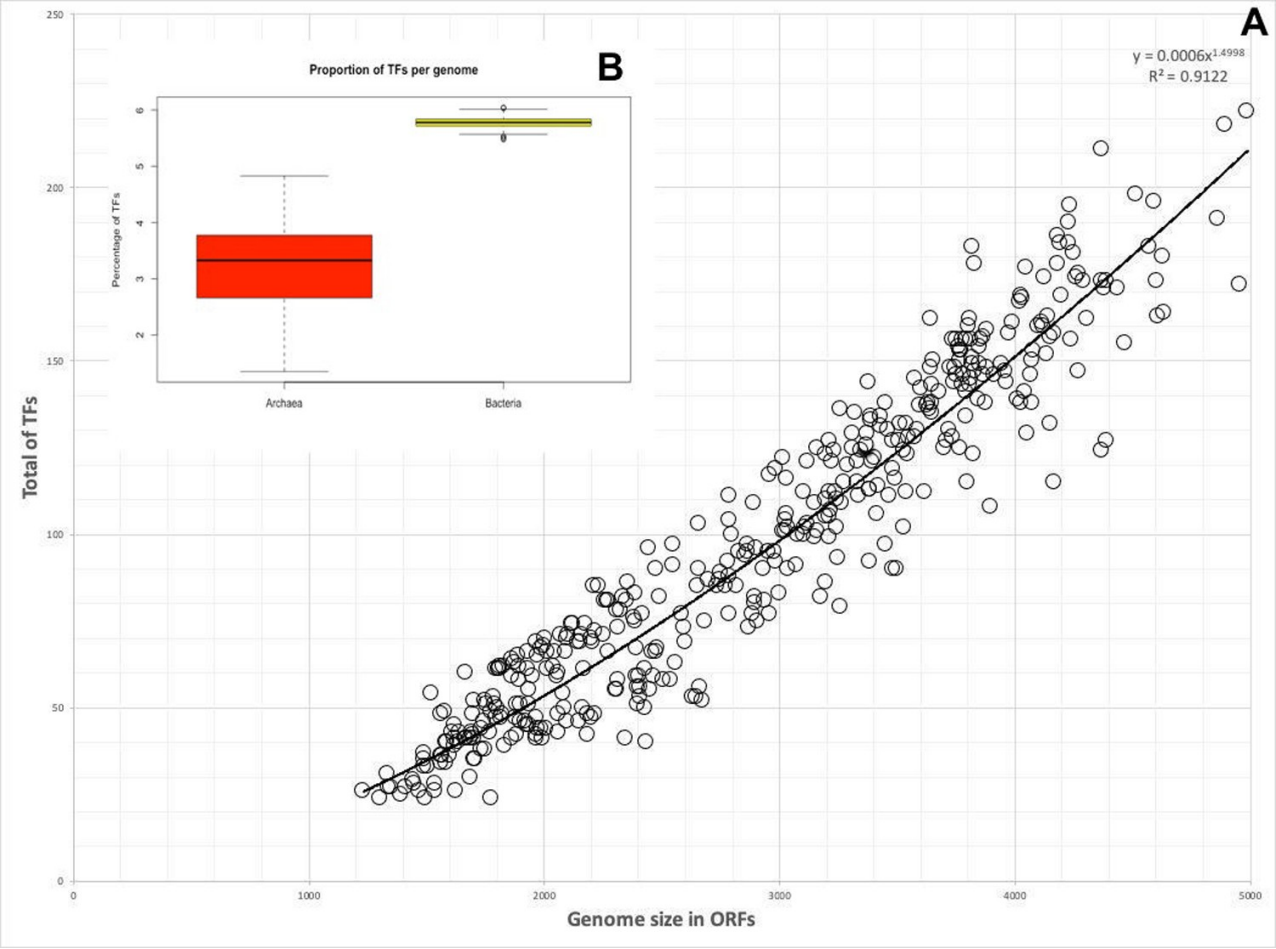
a) Abundance of TFs in Archaea as a function of genome size (open reading frames, or ORFs). On the y axis is the total TFs for each genome. Each dot corresponds to one genome. The power fit function (black line) and R^2^ are indicated (y = 0.0006 × 1.4998). b) Proportion of TFs in all the archaeal genomes and the normalized values of TFs in representative bacterial dataset. The distributions of TF fraction are different from each other (Wilcoxon test, P-value < 2.2e-16).

The repertoire of predicted TFs in archaeal genomes follows a power distribution, with R^2^ = 0.9122. This distribution has been previously described in bacterial TFs, suggesting that the correlation between genome size and number of TFs is similar in bacteria and archaea, i.e., small genomes contain a small number of TFs and large genomes contain a large proportion of TFs [7] (Fig 1). However, the proportion of protein-encoding genes devoted to regulate gene expression is lower in archaea than bacteria. In this regard, we found an average of 3.21 ± 0.69% TFs in the proteomes of archaea, in contrast to bacteria, with 5.77 ± 1.87% TFs per genome (Fig 1). To exclude a bias of overrepresentation of the 12466 bacterial genomes or an uneven sampling of genomes with different size ranges, we compare the proportion of TFs with equivalent subsets of bacterial genomes. To do this, we randomly sampled 415 bacterial genomes 1000 times each, obtaining the average of TF proportion each one and their statistical differences were evaluated. Wilcoxon’s test shows that the TF proportion in these equivalent groups are different (p-value < 2.2e-16). This finding suggests a smaller proportion of TFs in archaea than bacteria, even in organisms with similar genome size.

In this context, the extreme halophilic archaeon isolated from a sulfate saline soil in Turkmenistan, *Haloterrigena turkmenica* DSM 5511, exhibits the largest number of regulatory proteins predicted, with 222 TFs. The repertoire of TFs represents 4.99% of the total 4911 protein-encoding genes predicted in this organism. In contrast, the anaerobe hyperthermophilic archaea *Methanopyrus kandleri* AV19, isolated from from the sea floor at the base of a 2,000-m-deep “black smoker” chimney in the Gulf of California, was identified with the lowest repertoire of TFs. In this organism, 24 proteins were predicted as devoted to regulation of gene expression, representing 1.35% of the total of 1775 proteins associated with this archaeon.

On the basis of these predicted TFs, an apparent deficit of TFs was found in archaeal genomes compared with their bacterial counterpart. This deficit could be compensated with new classes of TFs present in archaeal genomes or alternative regulatory mechanisms, such as the RNA processing sites in methanogenic archaea associated with translational regulation [19] or small regulatory RNAs [20, 21]. Otherwise, it is probable that a bias exists in the dataset used as reference to identify the repertoire of TFs in archaea, mainly associated with bacterial ones.

### The repertoire of TFs shows different proportion of families

**Fig 2:**
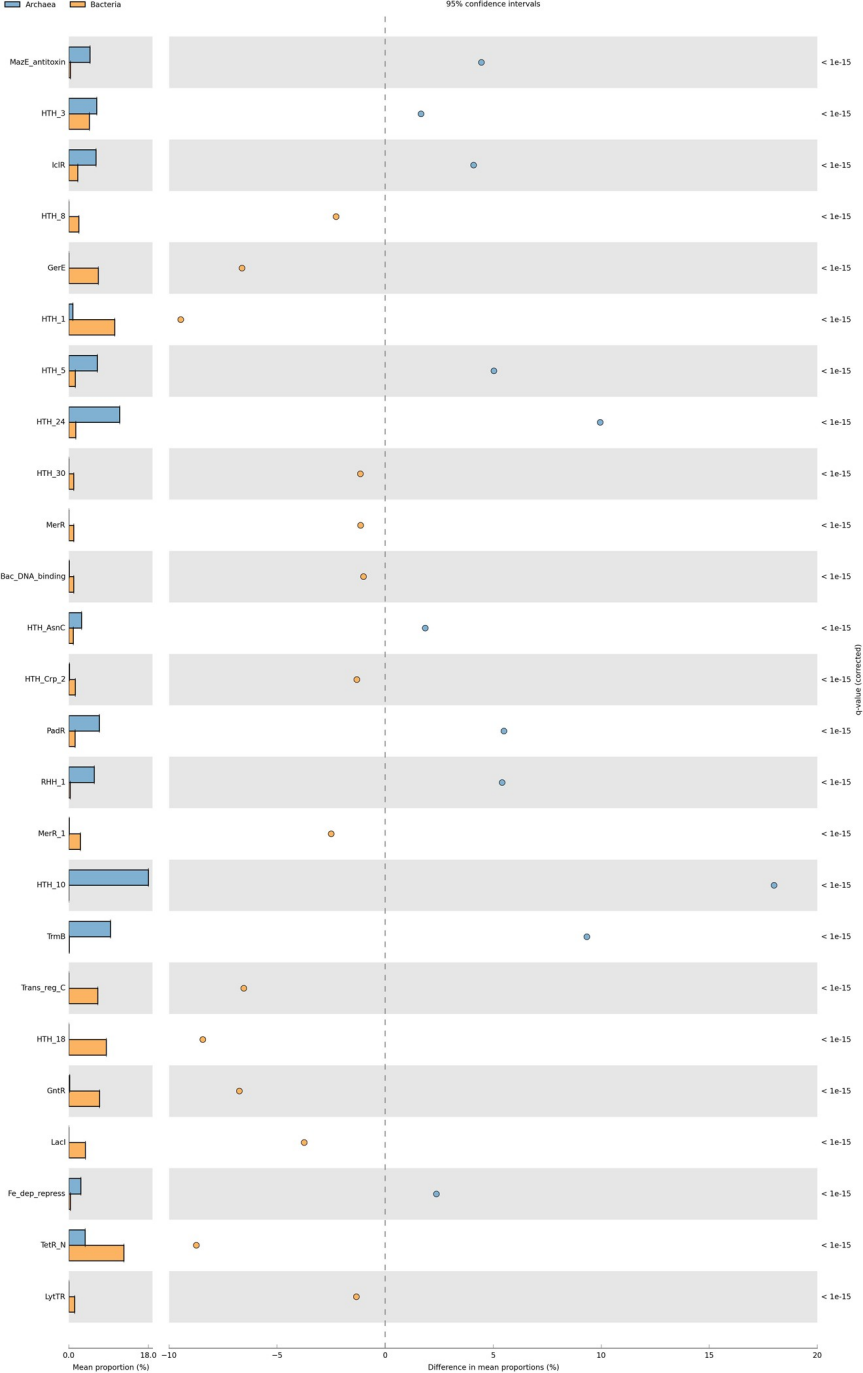
Proportion of families in archaea. The PFAM domains were evaluated to assess the existence of significant differences in their relative proportions. In orange are indicated the archaeal families and in blue the bacterial families. On the x axis is the mean proportion per family (%) and the difference in mean proportions (%). The protein families are in the y axis.

In general, proteins can be clustered into families based on sequence comparisons [14]. In order to determine the distribution of these families in archaea, the 40478 archaeal proteins were clustered into 62 different families, and their abundances were compared with the families identified in bacteria. From these, we found 11 families that are more abundant in archaea than in bacteria (Fig 2). These families include HTH_24, FFRP (PF13412), and TrmB (PF01978). For instance, the FFRP contains proteins described as global regulators, such as LysM of *S*. *solfataricus* that, in the absence or at low concentrations of lysine, activates the biosynthesis of this amino acid via the alpha-aminoadipate (AAA) pathway [22]; members of the Sugar-specific transcriptional regulator TrmB family of *Pyrococcus furiosus*, which inhibit transcription of the trehalose/maltose transport gene cluster (*malE* operon), and of the maltodextrin transport gene cluster (*mdxE* operon) [23] (Fig 2).

A total of 13 families were found as barely abundant in archaea in comparison to their bacterial counterparts, such as GerE, LytTR, and HTH_1 (LysR). Indeed, these families are the most common and abundant families in bacteria, suggesting that their low abundances in archaea could be a consequence of the expansion of alternative families with a large number of members in bacteria (Fig 2).

38 families were found with not significant differences in archaea and bacteria, such as PspC, MarR, and HTH_11. These families are involved in resistance to antibiotics (MarR, Penicillinase_R), cold shock (CSD), and phage shock responses (PspC). We consider that these families could have similar functions in archaea and bacteria because of their similar distributions; indeed, they are involved in the regulation of stress responses, such as antibiotic resistance or phage shock, among others.

### Archaeal and bacterial TFs show significant differences in size

**Fig 3:**
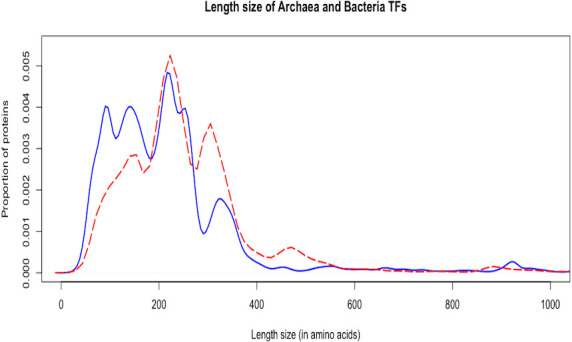
Sizes of TFs in archaea and bacteria. The continuous lines indicate archaeal TF data, and dashed lines show the bacterial ones (p-value < 2.2e-16).

It has been proposed that transcription regulation in archaea is chimeric, with proteins belonging to the basal transcriptional machinery that seem eukaryote-like, whereas proteins devoted to regulate, positively or negatively, gene regulation are bacterial-like [24]. In this regard, the identification of regulatory proteins in the archaeal organisms showed a large proportion of small proteins, in contrast to their bacterial counterpart (Fig 3); these small proteins included members of the FFRP family, such as LrpA/Trh7 in *H*. *salinarum* (Q9HQK1_Halsa) with 75 amino acids (aa), described as a leucine regulator [22] or Trh3 (Q9HP41_ alsa) of *H*. *salinarum* (with 137 aa), a dimeric protein involved in the regulation of leucine [22]; Lrs14 of *S*. *solfataricus*, a member of the TrmB family (LRS14_Sacs2) with 125 aa, involved in trehalose metabolism, blocking the TBP and TFB recruitment [25], and DtxR of *P*. *furiosus* (Q8U2I3_Pyrfu), with 133 aa, regulating the expression of genes involved in metal homeostasis [22]. Therefore, in order to determine if there is a difference in the size associated with the TFs in archaea versus bacterial TFs, 415 bacterial genomes 1000 times each were randomly sampled, and their statistical differences were evaluated, finding that the archaeal TFs are smaller than bacterial TFs (Wilcoxon’s test shows differences between both datasets, p-value < 2.2e-16). This finding correlates with the fact that 60% of the TFs in archaea are monodomain, 32% have two domains, and 8% of the proteins contain more than three domains. In contrast, in bacterial TFs, 41% are monodomain, 53.4% have two domains, and approximately 5% contain more than three structural domains.

### Comparison of the repertoires of TFs shows significant differences in the pIs

To determine the distribution of isoelectric points (pIs) of proteins identified as TFs in archaea, these proteins were compared against bacterial TFs. This analysis was done under the premise that proteins are the most insoluble, least reactive, and unstable at pHs close to their pI, and the pH of the majority of the cellular interior compartments is close to 7.5. Thus, this property could be the result of selection at the very early steps of evolution [26].

**Fig 4:**
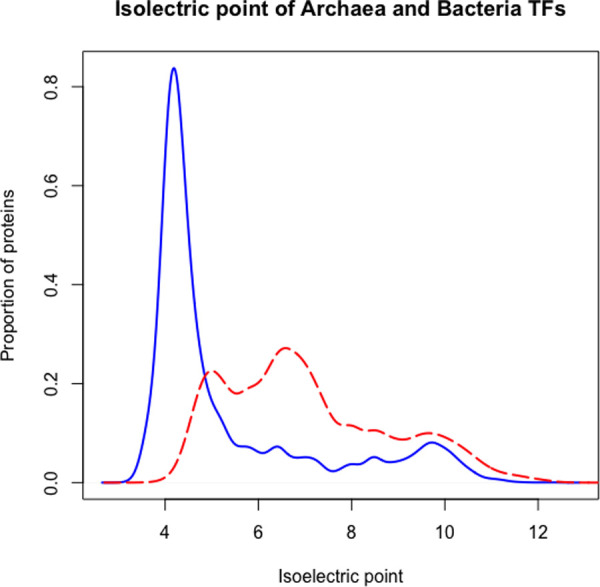
Isoelectric points associated with the TFs in archaea (continuous lines) and bacteria (dashed lines). (Wilcoxon test, p-value < 2.2e-16).

To evaluate the pI, the archaea genomes was compared against a random sampled of 415 bacterial genomes, 1000 times each (Fig 4). We found that the pI of archaeal TFs is bimodal, where the first peak corresponds to 4.0 and a second peak is close to 10.0. In contrast, bacterial TFs have different pIs; the first one corresponds to a pI of 5.0 and the second peak to 6.5. Wilcoxon’s test shows that the proportion are different (p-value < 2.2e-16). This difference suggests that archaeal TFs contain a major proportion of acidic amino acids in comparison to bacterial TFs. These archaeal proteins (with pI of 4.0) are mainly included in the HTH_10 family (5389 out of 7354), followed by the TrmB family (2067 out 3378), whereas the second peak, identified around pI of 10, is mainly associated with the HTH_24 (174 out 4121), followed by the ArsR family (153 out 2321), with a high proportion of basic amino acids.

In summary, our results show that archaeal TFs with an acidic pIs predominate over the TFs with an alkaline pI. In contrast, bacterial TFs are mainly associated with basic amino acids. This distribution could be a consequence of the differential composition of amino acids in different cellular domains and might be associated with environmental and ecological pressures. Therefore, this result is consistent with the analysis on bacterial proteomes, where the multimodal distribution of pI corresponds to the pK values of amino acid moieties [27, 28]

### Archaeal genomes encode a large proportion of TFs with enzymatic activities

**Fig 5:**
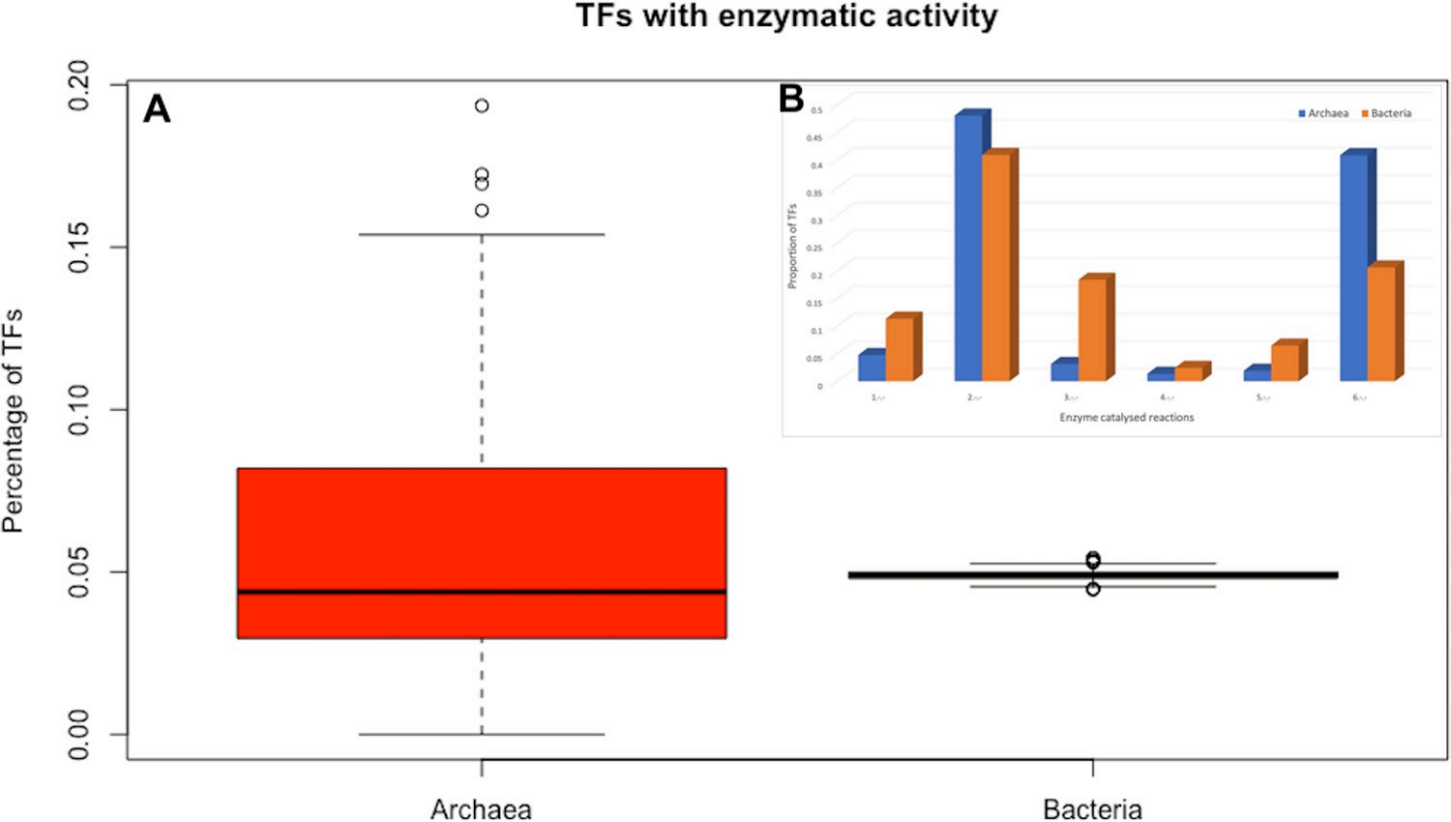
a) Proportion of enzymatic reactions associated to archaeal TFs and the normalized values of enzymatic reactions in representative bacterial dataset. The distributions of TF fraction are different from each other (Wilcoxon test, P-value = 0.00792). b) Proportion of the x axis is the catalytic activity (EC number) and on the y axis is the proportion of each activity.

In order to determine whether TFs in archaeal genomes have a high proportion of dual activities (enzyme and DNA binding), the repertoire of proteins devoted to gene regulation was compared against the TFs identified in bacterial genomes. To perform this evaluation, the set of archaea was compared against a random sampled of 415 bacteria genomes (1000 times each), and repertoires of proteins with catalytic activities in both datasets were compared. We found that 5.79% of the archaeal TFs were also devoted to enzymatic activities. In contrast, an average 4.9% of the bacterial TFs were associated with this function. In order to exclude a bias of overrepresentation of the 12466 bacterial genomes, we randomly sampled 415 bacterial genomes 1000 times each, obtaining the average of TFs with enzymatic activity each one and their statistical differences were evaluated. Wilcoxon’s test shows that the proportion in these equivalent groups are different, (p-value = 0.00792), indicating that TFs in archaea tend to be significantly smaller than the overall proteome (Fig 5).

In addition, we evaluated the distribution of enzymatic activities associated with the repertoire of TFs. From this, the EC:2.-.- Transferases and EC:6.-.- Ligases were more abundant in archaeal TFs but not in bacteria (Fig 5). For instance, the TFs WP_062266039 of *Methanoculleus bourgensis*, classified as a member of the MarR family, also contains a protein/riboflavin kinase activity (2.7.1.161), and WP_056934426 identified in *Thermococcus barophilus*, which performs a role in TF binding to DNA by a h-t-h domain, also exhibits a function of biotin-[acetyl-CoA-carboxylase] ligase (EC 6.3.4.15) [29]. In contrast, the EC:1.-.- Oxidoreductases, EC:3.-.- Hydrolases, EC:4.-.- Lyases, and EC:5.-.- Isomerases are more abundant in bacterial TFs in comparison to the archaeal regulatory proteins. We consider that the transferase and ligase activities predominantly identified in archaeal TFs could play a role as sensors in membranes, as peripheral membrane proteins or proteins anchored to membranes to posteriorly act as regulators, probably as those proteins described as two-component systems, not identified (so far) in archaea.

### Archaeal genomes encode a minor proportion of TFs involved in virulence activities, compared with bacterial genomes

**Fig 6:**
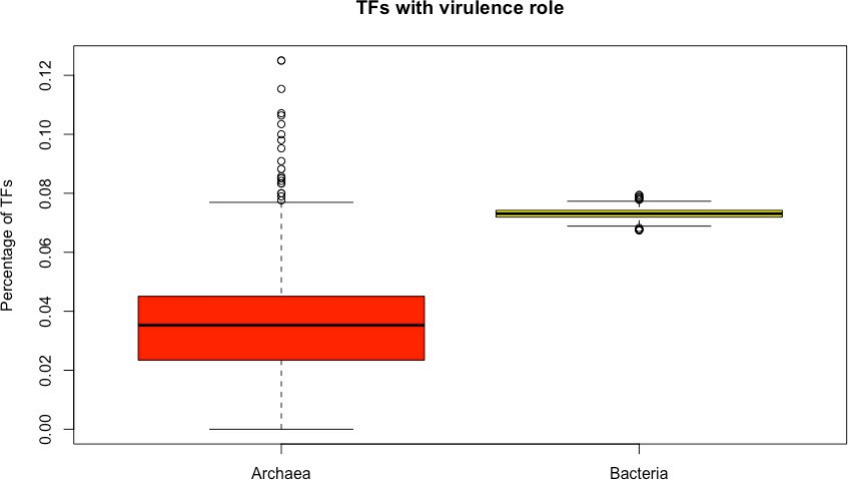
Proportion of TFs with virulence roles in archaea and the normalized values in representative bacterial dataset. The distributions of virulence fraction are different from each other (Wilcoxon test, p-value < 2.2e-16).

In order to determine the proportion of TFs with a role in virulence, the repertoire of predicted regulatory proteins in archaea was compared against the TFs identified in bacterial genomes. To achieve the evaluation, the set of TFs of the 415 archaeal genomes was compared against a random sampled of 415 bacteria genomes (1000 times each). From this analysis, we found that 3.6% of the TFs in archaea also have a homologous in the set of proteins deposited in VFDB, whereas in bacteria this set corresponds to an average of 7.3%; i.e., there is a low proportion of archaeal TFs devoted to virulence, in contrast to bacterial proteins. To validate this finding, we randomly sampled 415 bacterial genomes 1000 times each, obtaining the average of TFS with virulence roles each one and their statistical differences were evaluated. Wilcoxon’s test shows that the proportion in these equivalent groups are different, (p-value < 2.2e-16) (Fig 6). This result correlates with the fact that archaea are mainly described as free-living or opportunistic organisms, but not pathogens, suggesting that the role of these proteins could be associated with transport across the membrane, such as the Fe^2+^ transport system protein FeoA (WP_010875853) of *Methanothermobacter defluvii* or global regulators such as the h-t-h transcriptional regulator of *Archaeoglobus fulgidus* (WP_010879124). In this regard, the cell surface proteins that mediate bacterial attachment, cell surface carbohydrates and proteins that protect the bacterial cell, in addition to hydrolytic enzymes may contribute to the pathogenicity of the organism [30]. Thus, we suggest that TFs identified as homologous in this dataset could also contribute to the cell protection or associated with enzymatic activities, as previously described.

### Archaeal genomes encode more TFs with intrinsically disordered regions than bacterial genomes

**Fig 7:**
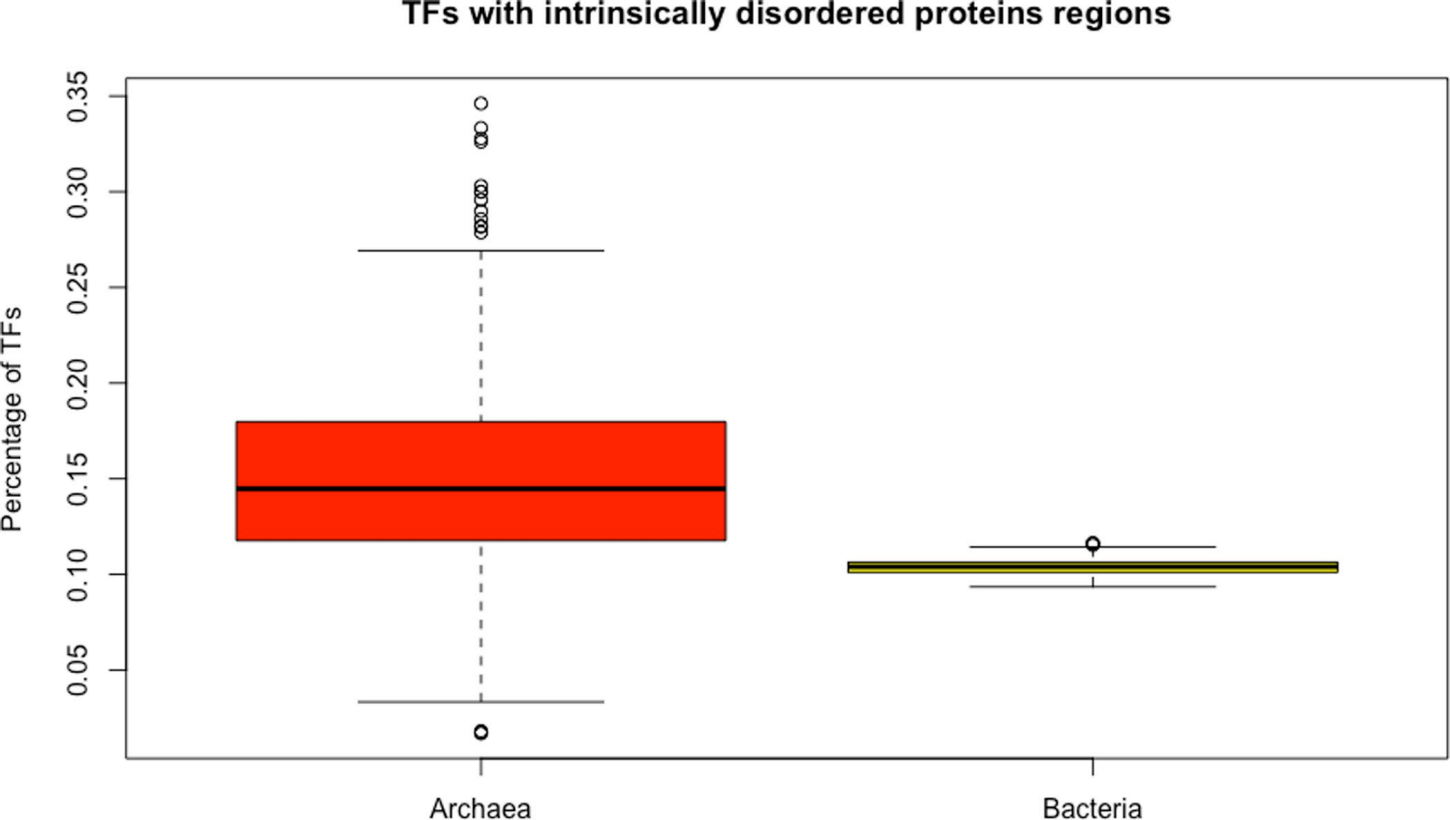
Proportion of TFs with disorder regions in archaea and the normalized values in representative bacterial dataset. The distributions are different from each other (Wilcoxon test, p-value < 2.2e-16).

Intrinsically disordered proteins (IDPs) have no single well-defined tertiary structure under native conditions; however, recent studies revealed that the content of IDPs in the nodes could modulate network topology, rewire networks, and change their interconnectivity, which is defined by its clustering coefficient [31]. Therefore, we determined the role of these proteins in archaeal TFs versus bacterial TFs. To do this, IUPred software was used, which allows identification of disordered protein regions that do not adopt a stable structure, depending on the redox state of their environment. From this analysis, we found that 15.2% of the total of TFs of archaea also contain an IDP region, in comparison to (an average of) 10.39% TFs with these structures in bacteria. The validation with the random sampling of 415 bacterial genomes 1000 times each, obtaining the average of TFS with disorder regions each one and their statistical differences were evaluated. Wilcoxon’s test shows that the proportion in these equivalent groups are different, (p-value < 2.2e-16) (Fig 7). For instance, WP_009486605 from the LuxR family in *Halobacteria*, was identified with nine regions identified as IDPs, along with two small proteins from the cold shock protein family, identified in *Halobacteria* and *Natrialbaceae* (WP_008418277.1 and WP_005578474.1). This slight abundance in IDPs in archaeal regulatory proteins suggests that archaeal TFs have more disordered regions than bacterial TFs. The role of these proteins could be directly related to the connectivity of the network, where more IDPs have been identified as hubs than end proteins, defined here as those that interact with just one partner [32]. Thus, we consider that the reconstruction of regulatory networks in archaea is fundamental to determining the role of these regions in the TFs.

## Conclusions

In this work, we evaluated the hypothesis that TFs in archaea and bacteria are homologous; however, their composition and functional role could be different as a consequence of evolutionary divergence. To this end, an exhaustive analysis identifying this class of proteins in 105 nonredundant archaeal genomes was performed. In a posterior step, a systematic comparison against the bacterial TFs was achieved. From this analysis, we identified an apparent deficit of TFs in archaea in comparison with their bacterial counterparts, which could be compensated with alternative regulatory mechanisms or new classes of TFs in archaeal genomes. We also suggest different abundances of families, such as TrmB and FFRP in archaea, and some of them that are absent in bacteria. A different proportion of dual activities in archaeal TFs versus bacterial TFs could also compensate for the apparent deficit of this class of proteins in archaea. Finally, we need to increase the number of proteins with experimental evidence. To date, there are few TFs (61) that have been experimentally characterized in the literature, mainly associated with just a few organisms, such as *H*. *salinarum*, with 22 TFs, followed by *P*. *furiosus* with six TFs and *S*. *solfataricus* and *Methanococcus jannaschii*, with three TFs each (S3 File). This information is relevant in the context of the development of analysis to predict regulatory proteins in these organisms to be experimentally characterized in the meantime.

## Supporting information

S1 File List of PFAM IDs associated to TFs with experimental evidences, retrieved from diverse databases. (TXT)

S2 File Prediction of TFs in archaeal and bacterial genomes. (DOCX)

S3 File List of TFs with experimental evidence in archaea. (XLSX)
