# Supplementary material for: Comparative genomics of DNA-binding transcription factors in archaeal and bacterial organisms
Source: PLoS One. 2021 Jul 2;16(7):e0254025. doi: 10.1371/journal.pone.0254025 (PMC8253408; doi:10.1371/journal.pone.0254025)
Supplement: S2 File — (DOCX) [file pone.0254025.s002.docx]

**S2 File. List of TFs with experimental evidence in archaea.**

| **Uniprot ID** | **Protein name** | **Organism** | **Family** | **PFAM ID(s)** | **Function** |
| --- | --- | --- | --- | --- | --- |
| Q6V4G9_METVO | HrsM | *Methanococcus voltae* | LysR | PF00126, PF03466 | Hydrogenase gene regulator, selenium dependent |
| GLPR_HALVD | GlpR | *Haloferax volcanii* | DeoR | PF00455, PF08220 | Transcriptional repressor of genes encoding both fructose and glucose metabolic enzymes, such as phosphofructokinase (PFK) or 2-keto-3-deoxy-D-gluconate kinase (KDGK) during growth on glycerol. Required for the glycerol-mediated repression of pfkB-specific transcripts, but not needed for the high levels of the pfkB-specific transcript present when cells are grown on fructose. |
| C5A3E5_THEGJ | HypF | *T. gammatolerans* | zf-HYPF | PF00708, PF17788, PF00814, PF01300, PF07503 | Acylphosphatase activity, carboxyl- or carbamoyltransferase activity, double-stranded RNA binding and zinc ion binding |
| HMFA_METFE | HmfA | *Methanothermus fervidus* | HMF | PF00808 | Archaeal histone; increases the resistance of DNA to thermal denaturation |
| HMFB_METFE | hmfB | *M. fervidus* | HMF | PF00808 | Archaeal histone; increases the resistance of DNA to thermal denaturation |
| Q8U030_PYRFU | PhrA | *P. furiosus* | ArsR | PF01022 | Negatively regulates its own transcription; binds to a 46-base pair sequence that overlaps the transcriptional start site of its own promoter |
| O28971_ARCFU | AF_1298 | *Archaeoglobus fulgidus* | TrmB | PF01022, PF01978 | Thermal shock protein |
| LYSM_SULSO | LysM | *S. solfataricus* | FFRP | PF01037 | In the absence of or at low concentrations of lysine, activates the biosynthesis of this amino acid via the alpha-aminoadipate (AAA) pathway |
| PTR1_METJA | Ptr1 | *M. jannaschii* | FFRP | PF01037 | Participates in positive as well as negative regulation of transcription; binds to its own promoter |
| Q9HNI1_HALSA | Lrp/Trh4 | *H. salinarum* | FFRP | PF01037 | Leucine regulator |
| Q9HQ13_HALSA | AsnC | *H. salinarum* | FFRP | PF01037 | Regulators of the AsnC family control a variety of cellular processes, including energy and core metabolism, amino acid metabolism, ATP biosynthesis, DNA repair and recombination, bacterial persistence, and virulence |
| Q9HQ33_HALSA | Trh6 | *H. salinarum* | FFRP | PF01037 | Leucine regulator |
| Q9HQK1_HALSA | LrpA/Trh7 | *H. salinarum* | FFRP | PF01037 | Leucine regulator |
| REG7_PYRFU | lrpA | *P. furiosus* | FFRP | PF01037 | Negatively regulates its own transcription; binds to a 46-base pair sequence that overlaps the transcriptional start site of its own promoter |
| REG7_PYRFU | lrpA | *P. furiosus* | FFRP | PF01037 | Negatively regulates its own transcription; binds to a 46-base pair sequence that overlaps the transcriptional start site of its own promoter |
| PTR2_METJA | Ptr2 | *M. jannaschii* | FFRP | PF01037, PF13404 | Participates in positive as well as negative regulation of transcription; binds to its own promoter |
| Q9HP41_HALSA | Trh3 | *H. salinarum* | FFRP | PF01037, PF13404 | Leucine regulator |
| REG6_PYRHO | Fl11 | *Pyrococcus horikoshii* | FFRP | PF01037, PF13404 | Repression of transcription of genes involved in ATP synthesis, transmembrane transport, translation, and DNA synthesis |
| ARNR_SULAC | ArnR | *Sulfolobus acidocaldarius* | MarR | PF01047 | Involved in regulation of archael gene expression; activates flaB transcription upon nutrient starvation by acting on the flaB promoter |
| Q9HME7_HALSA | Idr1 | *H. salinarum* | Fe dep repress | PF01325 | IdeR is a metal-sensing transcription factor that regulates free iron concentration |
| Q9HR69_HALSA | Idr2 | *H. salinarum* | Fe dep repress | PF01325 | IdeR regulates free iron concentration |
| MSRA_METAC | MsrA | *Methanosarcina acetivorans* | ArsR | PF01625 | Repair enzyme for proteins that have been inactivated by oxidation; catalyzes the reversible oxidation-reduction of methionine sulfoxide to methionine in proteins |
| Q9HS14_HALSA | PhoU | *H. salinarum* | PhoU | PF01895 | Regulation of phosphate uptake |
| ALBA_METJA | AlbA | *M. jannaschii* | Alba | PF01918 | Maintains the structural and functional stability of RNA and ribosomes |
| ALBA_PYRHO | AlbA | *P. horikoshii* | Alba | PF01918 | Maintains the structural and functional stability of RNA and ribosomes |
| ALBA_THEVO | AlbA | *Thermoplasma volcanium* | Alba | PF01918 | Maintains the structural and functional stability of RNA and ribosomes |
| ALBA1_SACS2 | AlbA1 | *S. solfataricus* | Alba | PF01918 | Plays a role in maintaining the structural and functional stability of RNA and ribosomes. |
| ALBA1_SULSH | AlbA1 | *Sulfolobus shibatae* | Alba | PF01918 | Maintains the structural and functional stability of RNA and, perhaps, ribosomes |
| ALBA2_AERPE | AlbA2 | *Aeropyrum pernix* | Alba | PF01918 | Plays a role in maintaining the structural and functional stability of RNA and, perhaps, ribosomes |
| ALBA2_SACS2 | AlbA2 | *S. solfataricus* | Alba | PF01918 | Plays a role in maintaining the structural and functional stability of RNA and, perhaps, ribosomes |
| LRS14_SULSO | Lrs14 | *S. solfataricus* | TrmB | PF01978 | Blocks TBP and TFB recruitment |
| C5A5A4_THEGJ | RibK | *Thermococcus gammatolerans* | HTH_27 | PF01982, PF13463 | Catalyzes the CTP-dependent phosphorylation of riboflavin (vitamin B2) to form flavin mononucleotide (FMN) |
| NRPR2_METMA | NpRII | *Methanosarcina mazei* | NrpR | PF01995 | Involved in nitrogen regulation; under nitrogen sufficiency, represses transcription of the nifH and the glnk1 promoters, via formation of a complex with the global nitrogen regulator NrpRI |
| Q9HQ80_HALSA | Trh2 | *H. salinarum* | TrkA | PF02080 | Leucine regulator |
| Q8U2I3_PYRFU | PF0851 | *P. furiosus* | Fe dep repress | PF02742, PF01325 | Regulates the expression of genes involved in metal homeostasis. |
| Q9HRV3_HALSA | TroR | *H. salinarum* | Fe dep repress | PF02742, PF01325 | Iron-dependent transcription repressor |
| Q9HRU8_HALSA | SirR | *H. salinarum* | DtxR | PF02742, PF01325, PF04023 | SirR functions as a divalent metal cation-dependent transcriptional repressor which is widespread |
| Q9HJU1_THEAC | Ta0872 | *Thermoplasma acidophilum* | FeoA | PF02742, PF04023 | Iron-dependent transcription repressor |
| F9VNA8_SULTO | WrbA | *Sulfurisphaera tokodaii* | FMN_red | PF03358 | NAD(P)H--quinone oxidoreductase WrbA (EC 1.6.5.2) |
| GVPE1_HALSA | GvpE | *H. salinarum* | PadR | PF03551 | Can play a structural or regulatory role in the synthesis of gas vesicles |
| Q9HSF4_HALSA | RosR | *H. salinarum* | PadR | PF03551 | Modulates the expression of genes that encode proteins involved in repairing cell damage from extremely high levels of reactive oxygen species (ROS) |
| Q9HRB9_HALSA | Prp2 | *H. salinarum* | PhoU | PF04014, PF01895 | Phosphate regulatory protein homolog; also has SpoVT/AbrB domain in the N terminus |
| D4GPG1_HALVD | NarO | *H. volcanii* | HTH_10 | PF04967 | Activates transcription of denitrifying genes under anaerobic conditions |
| D4GQ08_HALVD | DmsR | *H. volcanii* | HTH_10 | PF04967 | Regulator of nitrate-reductase and dimethyl sulfoxide (DMSO) reductase |
| DMSR_HALSA | DmsR | *H. salinarum* | HTH_10 | PF04967 | Participates in activation of the expression of the gene dmsEABCD, related to DMSO |
| Q9HNX8_HALSA | KaiC | *H. salinarum* | KaiC | PF06745 | Circadian regulator |
| GVPD2_HALSA | GvpD2 | *H. salinarum* | GvpD | PF07088 | Energy-requiring process, such as assembly of gas |
| B2CQV6_SULS9 | CopR | *S. solfataricus* | TRASH | PF08394 | Transcriptional regulator with copper detection and acts as a repressor for self-regulation and for an alleged CopA copper efflux system |
| Q9HQG1_HALSA | VNG_1179C | *H. salinarum* | FFRP | PF08394, PF13404 | Interacts selectively and noncovalently with DNA of a specific nucleotide composition |
| NRPR_METJA | NrpR | *M. jannaschii* | NrpR | PF08461, PF01995 | Global nitrogen regulator |
| NRPR1_METMA | NpRI | *M. mazei* | PII | PF08461, PF01995 | Nitrogen regulation; under nitrogen sufficiency, binds to the nifH and the glnk1 promoters, leading to repression of the transcription of the genes |
| NRPR_METMP | NrpR | *Methanococcus maripaludis* | NRD1 | PF08461, PF01995, | Transcriptional repressor of nitrogen fixation and assimilation genes |
| Q9HNM3_HALSA | Hlx1 | *H. salinarum* | HalX | PF08663, PF00072 | Regulation of phosphorelay signal transduction system |
| Q9HHM8_HALSA | arcR | *H. salinarum* | IclR | PF09339, PF01614 | Negative regulation of cellular transcription; DNA-dependent |
| TMBL1_PYRFU | TrmBL1 | *P. furiosus* | TrmB | PF11495, PF01978 | Global transcriptional repressor of the maltodextrin transport gene cluster (mdxE operon) and most likely of all genes encoding glycolytic enzymes |
| TMBL2_THEKO | TrmBL2 | *Thermococcus kodakaraensis* | TrmB | PF11495, PF01978 | Genome architecture and transcription repression |
| TRMBR_HALSA | TrmB | *H. salinarum* | TrmB | PF11495, PF01978 | Expression of genes functioning in central metabolism; acts as both a transcriptional activator and a repressor in response to carbon source availability |
| TRMBR_PYRFU | TrmB | *P. furiosus* | TrmB | PF11495, PF01978 | Inhibits transcription of the trehalose/maltose transport gene cluster (malE operon) and of the maltodextrin transport gene cluster (mdxE operon) |
| TRMBR_THELN | TrmB | *Thermococcus litoralis* | TrmB | PF11495, PF01978 | Inhibits transcription of the trehalose/maltose transport gene cluster (malE operon) |
| Q9HQB6_HALSA | VNG1237C | *H. salinarum* | FFRP | PF13404 | Leucine regulator |
| ARNR1_SULAC | ArnR1 | *S. acidocaldarius* | HTH_45 | PF14947 | Involved in regulation of archaellar gene expression; activates flaB transcription upon nutrient starvation by acting on the flaB promoter |
| BAT_HALSA | Bat | *H. salinarum* | HTH_10 | PF15915, PF13185, PF04967, PF13426 | Involved in activating bop (bacterioopsin) and brp gene expression at low-oxygen tension, which naturally occurs in stationary phase |
